# Carbon Nanotubes and Graphene Materials as Xenobiotics in Living Systems: Is There a Consensus on Their Safety?

**DOI:** 10.3390/jox13040047

**Published:** 2023-12-01

**Authors:** David Gendron, Grzegorz Bubak

**Affiliations:** 1 Kemitek, Cégep de Thetford, 835 Rue Mooney, Thetford Mines, QC G6G 0A5, Canada; 2 Institute of Physical Chemistry, Polish Academy of Sciences, 01-224 Warsaw, Poland; gbubak@ichf.edu.pl

**Keywords:** carbon nanotubes, graphene, toxicity, xenobiotics, composites, biocompatibility

## Abstract

Carbon nanotubes and graphene are two types of nanomaterials that have unique properties and potential applications in various fields, including biomedicine, energy storage, and gas sensing. However, there is still a debate about the safety of these materials, and there is yet to be a complete consensus on their potential risks to human health and the environment. While some studies have provided recommendations for occupational exposure limits, more research is needed to fully understand the potential risks of these materials to human health and the environment. In this review, we will try to summarize the advantages and disadvantages of using carbon nanotubes and graphene as well as composites containing them in the context of their biocompatibility and toxicity to living systems. In addition, we overview current policy guidelines and technical regulations regarding the safety of carbon-based nanomaterials.

## 1. Introduction

**Figure 1:**
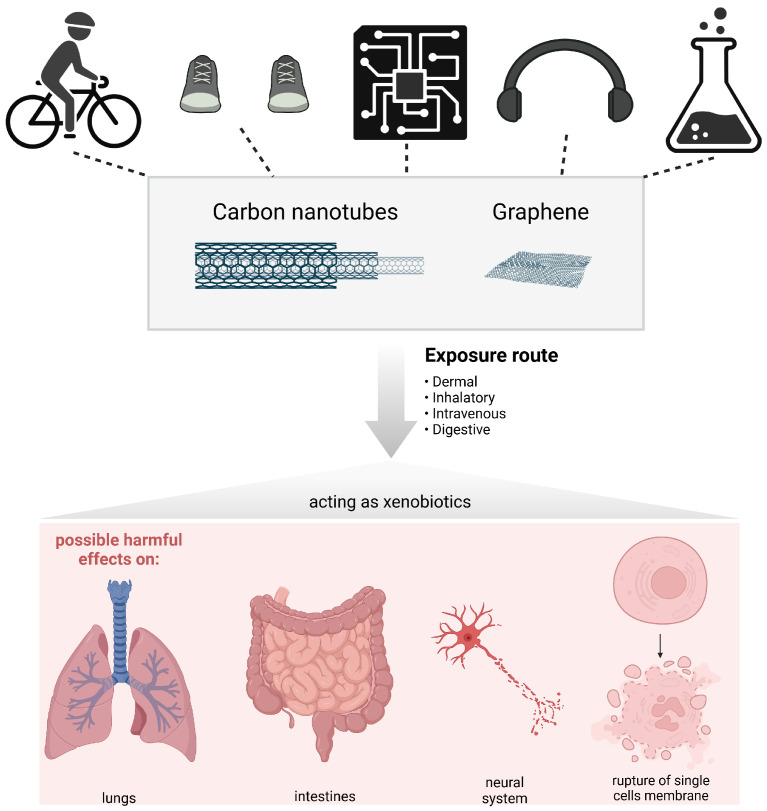
Schematic overview of this review. Carbon-based materials as carbon nanotubes and graphene are present in industrial products and applications, such as microelectronics, chemicals, bike tires, sportswear or helmets, as well as in consumer electronics such as headphones. From the majority of these materials, graphene and CNT can be released during or after the products’ lifecycle, acting as a xenobiotic when uptaken by living organisms, which can have potential adverse effects.

In recent years, the utilization of nanomaterials in a plethora of technological applications has raised concerns about their safety in living systems [1]. More specifically, the presence of carbon-based nanomaterials has sprung the investigation of their behavior in cells, organs and organisms as well as their repercussions on the environment. In this regard, carbon nanotubes and graphene can be regarded as xenobiotics, i.e., chemical compounds to which a biological system is exposed to that are exogenous to the normal metabolism of that system. Therefore, it is of critical importance to study the effect of the presence of carbon nanomaterials in biological systems as their use is now becoming available on a larger scale (present in a least 100 commercial product (CNTs) and produced at an annual rate of 20,000 tons (CNTs) and 4000 tons (graphene), respectively) [2]. However, as mentioned, CNTs and graphene pose a serious potential occupational health and safety hazard. Indeed, some CNTs are similar in shape to asbestos and were found to have a similar bio-persistence [3]. Several studies have shown that the needle-like fiber shape of some CNTs may lead to mesothelioma, fibrosis, and mesothelial proliferation [4]. As for graphene, it has been found that graphene interacts in similar ways as smaller stacked polycyclic aromatic hydrocarbons (PAH) [5]. Moreover, micro-sized graphene sheets have been reported to enter cells through spontaneous membrane penetration [6], thus enhancing its bio-persistence. Therefore, this review aims to discuss the aspects of carbon nanotubes and graphene as xenobiotics in living systems regarding their inherent safety (Figure 1). More specifically, the general physicochemical properties of graphene and CNTs are reviewed as well as their methods of preparation and characterization approaches employed for their analysis. Their exposure routes are briefly mentioned and their safety for humans are reviewed. At last, the actual guidelines and regulation concerning their safety are described.

## 2. Properties and Applications of Carbon Nanotubes and Graphene Materials

Carbon nanotubes (CNTs) and graphene are nano-sized materials and allotropes of carbon, such as diamond, graphite, amorphous carbon and fullerene. Carbon nanotubes were first discovered by Sumio Iijima in 1991 [7]. CNTs consist of a cylindrical molecule incorporating a hexagonal arrangement of sp^2^-hybridized carbon atoms. CNTs possess electronic, physical, thermal and mechanical properties that make them very sought after in the fields of nanotechnology, electronics and biomedicine to name only a few. Carbon nanotubes can be classified into two main categories: (1) single-walled carbon nanotubes (SWCNTs) or (2) multi-walled carbon nanotubes (MWCNTs). SWCNTs consist of a single layer of graphene with a diameter varying from 1 to 5 nm. On the other hand, MWCNTs consist of various layers of graphene sheets rolled with a diameter varying from 2 to 50 nm [8]. Chirality is an important aspect concerning CNTs as it dictates the resulting electronic properties. It is possible to classify CNTs into three types, chiral, armchair and zigzag, depending on the chiral vector (Ch) and chiral angle (θ) [9].

The principal methods carbon nanotubes production are the (1) arc discharge method. This method consists of arc-vaporizing two carbon rods carefully aligned in the presence of an inert gas atmosphere, such as helium or argon, at a pressure of 50 to 700 mbar. The carbon rod is then evaporated via a direct current (50–100 A), creating a discharge between the electrode and generating the CNTs [10]. (2) The laser ablation method. here, a pulsed laser is used to vaporize a graphite target located in an oven at 1200 °C. An inert atmosphere is used (He or Ar) at a pressure of 500 Torr, and arc discharge is initiated in the presence of a catalyst (Ni:Y) [11]. This method is primarily used for the preparation of SWCNTs. (3) The chemical vapor deposition method. This method is carried out in a two-steps process. First, a catalyst is deposited on a substrate followed by nucleation via chemical etching or thermal annealing. Second, the carbon source is converted to the atomic level using plasma, enabling the carbon to diffuse towards the substrate and allowing the growth of CNTs [12]. (4) The flame synthesis method. In this method, the SWCNTs are formed in a flame environment using hydrocarbon-based fuels together with a small aerosol metal catalyst (i.e., acetylene/oxygen/argon flame under 50 Torr) [13]. Lastly, (5) the silane solution method. The CNTs are prepared using a silane solution in which the substrate is carbon paper immersed in a metal catalyst solution (Co:Ni). A feedstock gas (ethylene) is then passed through the substrate, leading to the formation of CNTs [14]. Purification of carbon nanotubes can be achieved by air oxidation, acid reflux, steam or surfactant treatments [15,16,17].

In terms of the characterization of the carbon nanotubes in biological samples, several analytical approaches can be utilized to access their properties and evaluate their degree of functionalization [18]. For example, Raman spectroscopy is a useful method to rapidly screen the presence of SWCNTs. Transmission electron microscopy (TEM) allows for the detailed analysis of CNT structures [19]. Scanning electron microscopy (SEM) enables an overview analysis of sample structures [20]. At last, thermogravimetric analysis (TGA) provides information about the relative abundance of residual catalyst particles. Depending on the field of application, key aspects such as vibrational, thermal, mechanical properties as well as their electronic structure can be evaluated [21].

**Figure 2:**
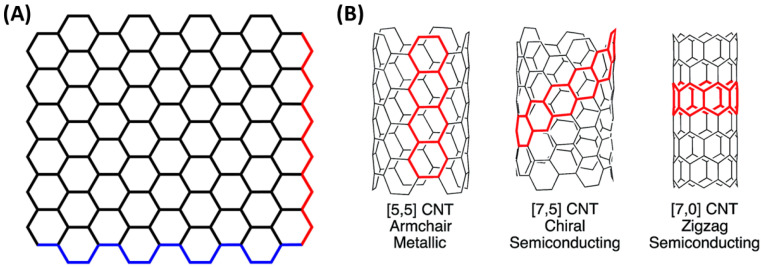
(**A**) Structure of graphene: armchair (blue) and zigzag (red), redrawn from [26]; (**B**) armchair, chiral or zigzag CNTs. Reproduced with permission from [27] from the Royal Society of Chemistry.

Graphene is often described as a one-atom-thick planar sheet of sp2-bonded carbon atoms which are packed into a honeycomb crystal structure (Figure 2). It is important to point out that a few layers of graphite are also considered graphene (≤10 layers). The inter-layer of crystalline graphene has been found to be 0.355 nm, primarily due to inter-planar bonding. Graphene is an excellent candidate for a lot of applications as it possesses a high intrinsic mobility, high thermal conductivity, and optical transmittance [22,23]. Graphene was first discovered in 2004 by Andre Geim and Konstantin Novoselov, but its structure was unambiguously reported [24]. Graphene layers have dimensions of 2 to 10 nm, depending on the method used to produce them [25].

Graphene can be prepared using two main approaches, namely a top-down approach or a bottom-up approach [26,28]. In the top-down approach, the mechanical exfoliation method is the best known. This method consists of using “Scotch tape” to peel off graphene layers from a graphite crystal [24]. This method leads to high-quality graphene with the film size ranging from 5 to 10 µm. However, this method is not suitable for mass production. Other top-down approaches include graphite intercalation, nanotube slicing (opening of carbon nanotubes), pyrolysis, reduction of graphite oxide (GO), electrochemical exfoliation, sonication and ball milling [29]. Concerning the bottom-up approach, graphene can be prepared by growth from metal-carbon melts, epitaxial growth on SiC, the dry ice method and chemical vapor deposition (a scalable method) [29]. Of these methods, the liquid exfoliation of graphite using N-methyl pyrrolidone is the method of choice as it can lead to defect-free monolayers of graphene [30]. Surfactants such as sodium dodecylbenzene or sodium chlorate sulfonate can also be used to exfoliate graphene in water [31]. Various characterization techniques can be used to obtain critical information about graphene flakes. More specifically, imaging methods such as atomic force microscopy (AFM), scanning electron microscopy (SEM) and high-resolution transmission electron microscopy (HR-TEM), combined or not, are preferred techniques to characterize the dimensions and physical aspects of the graphene layers [32]. More specifically, fluorescence quenching microscopy can be used to image the microstructure in graphene and graphene oxide flakes via dye-coated GO [33]. AFM (tapping mode) is employed to determine the thickness of the graphene layer at the nanometer scale as well as its mechanical properties [34]. In the case of graphene, TEM can be used to resolve the atomic features of the material, such as defects, atomic arrangement, and presence of contaminants [35]. At last, Raman spectroscopy is a useful technique to analyze the 2D and G bands present in most carbon allotropes such as graphene and CNTs. The identification of these bands provides information about the number of layers, the presence of defects, the effect of strain and doping concentration as well as the effect of temperature [36].

It is relevant to point out that both CNTs and graphene can be chemically functionalized to correspond to the desired biomedical application end properties. In this regard, several reviews have been published on the strategic approaches for both CNT and graphene materials [37,38,39,40]. Typical covalent methods include diazotization, cycloaddition, hydrogenation, fluorination, esterification, amidation, ion functionalization, organometallic reactions and electrophilic addition. Non-covalent methods feature surfactant and polymer wrapping, metal deposition, polymer encapsulation and adsorption. The main sought after properties of carbon nanotubes and graphene can be categorized as follows: charge transport properties, optical properties, mechanical properties and thermal properties [41,42,43]. More specifically for CNTs, SWCNTs show resistivity within the range from 5.1 × 10${}^{-6}$ to 1.2 × 10${}^{-4}$ ohm · cm [44]. Due to the chirality, CNTs can be metal or a semiconductor depending on how the free electron is delocalized over all the carbon atoms. SWCNTs are usually p-type semiconductors. In terms of mechanical properties, CNTs possess a Young’s modulus ranging from 270 to 950 GPa together with a tensile strength varying from 11 to 63 GPa [45]. TEM and AFM can be used in combination to determine the axial and radial deformation of the CNTs. Concerning the thermal properties of the CNTs, they were reported to possess a thermal conductivity over 3000 W/K · m at room temperature [46]. The phonon-active mode, the lengths of the free path for the phonon and the boundary surface scattering are factors influencing the thermal conductivity in CNTs. Consequently, these properties are also influenced by the diameter and length of the tubes, as well as the atomic arrangement, impurities, and structural defects. Mechanically exfoliated graphene has been reported to reach a high-charge mobility of over 200,000 cm${}^{2}$V${}^{-1}$s${}^{-1}$ in a transistor [47]. This high electronic conductivity is due to the very high quality (low defect density) of the graphene crystal. In terms of optical properties, graphene also showed 2.3% light absorption in the visible range for a monolayer [48]. Interestingly, the absorption of light was found to increase with the number to layers. Furthermore, the optical transition of graphene can be tuned by changing the Fermi energy, i.e., modifying the electrical gating of the optoelectronic devices [49]. Concerning thermal and mechanical properties, graphene has been reported to possess a thermal conductivity of 3000 W/K · m, a fracture strength of 130 GPa, a Young’s modulus of 1 TPa and a theoretical specific surface area of 2600 m${}^{2}$g${}^{-1}$ [46,50,51]. The thermal conductivity was attributed to the presence of C–C covalent bonds and phonon scattering. Compressive and tensile strength of graphene layers can be determined by AFM and Raman spectroscopy by monitoring the change in the G and 2D peaks under applied stress [52].

Concerning the role of CNTs and graphene, they are found in a variety of fields, namely electronic applications (field-effect transistors, sensor, supercapacitor, actuator) [53,54], clean energy devices (batteries and hydrogen storage) [55,56], and nanocomposites [8,57,58,59,60,61], owing to their charge transport properties, optical properties and high electrical conductivity. There application in the biomedical field is also studied (e.g., biosensors, imaging, drug delivery, cancer therapy, tissue engineering, etc.) [62,63,64,65,66,67]. For instance, the thermal properties of CNTs were used in medicine to enhance the efficacy of cancer treatment by selective cancer cell hyperthermia [68]. For example, graphene can be used as a biosensor based on its surface fluorescence quenching of specific target molecules [69]. Graphene and CNTs are now widely present in many applications. However little is known about their potential health and safety impacts towards humans and the environment. This brings the question about the exposure of living organisms to the presence of such nanomaterials. The exposure to graphene and CNTs can happen to workers (fabrication, transferring to containers, maintenance), consumers (composite materials, wear and tear, disposal), and patients (drug delivery systems, dermal contact and inhalation) during the material’s life cycle [70]. Therefore, the main exposures routes to CNTs are (1) from inhalation, (2) dermal contact, and (3) digestion. Inhalation of CNTs (both SWCNTs and MWCNTs) has been substantially investigated by several groups in workplaces and laboratories [70]. In this regard, the respiratory system is of utmost importance as it receives the entire cardiac output. Thus, the respiratory system can be subjected to secondary exposure to CNTs. Indeed, the upper airway is composed of the nose, mouth, pharynx and larynx. Likewise, the lower airways are composed of the trachea and bronchi as well as the pulmonary alveoli [71]. The pulmonary alveoli are the site of gaseous exchange (between air and blood) and contain over 300 million alveoli (about 140 m^2^). As reported, CNTs in the air form agglomerates and can be deposited in the lungs. For instance, the presence of CNTs via airborne exposure was measured by aerosol concentration [72] during the preparation of HiPco carbon nanotubes. HiPco SWCNTs are prepared in a unique way by decomposition of Fe(CO)${}_{5}$ in the presence of a continuous flow of CO [73]. This method of production leads to high-quality CNTs. Then, the filter samples can be analyzed by SEM to evaluate their sizes. Similarly, scanning transmission electron microscopy (STEM) was used to analyze tube structure and the presence of ropes and clumps [74]. It is worth mentioning that particle identification is critical to detect the source of the particles and the processes related to its formation. In this regard, TEM analysis provides information about the form (spherical or fiber), agglomerates or clusters. The airborne particle concentration depends on the particle characteristics, how it is measured and the working conditions. In terms of dermal exposure to CNTs, the available data is limited. A study by Maynard et al. reported CNT deposition on cotton gloves during the production of SWCNTs (finger and palm exposure) [72]. The authors found that the choice of appropriate gloves (i.e., using latex or nitrile instead of cotton) should minimize dermal exposure. As stated earlier, it is possible that some consumer products (plastic, sporting equipment, electronic equipment, textile) as well as medical devices (internal exposure, drug delivery, contrast agents) include CNTs. However, at this point, no study concerning the exposure of CNTs in these types of devices has been reported. Concerning graphene, the main exposures routes are similar to CNTs: (1) accidental ingestion, (2) inhalation, and (3) dermal exposure (i.e., consumer exposition through the skin caused by damage of a wearable graphene-based product [75]). However, the toxicity of graphene and graphene-derived materials depends primarily on their shape, dimension, number of layers, surface chemistry and behavior [76]. These physicochemical properties influence the biodistribution of graphene. For instance, a study of graphene production by CVD (chemical vapor deposition) in the workplace showed no measurable exposure risk to airborne graphene particles during the cleaning of the reactors [77]. The effect of graphene and CNTs in living systems (in vivo and in vitro toxicity) are discussed in the following sections.

## 3. Our Current Understanding of the Safety of Carbon Nanotubes and Graphene Materials

**Figure 3:**
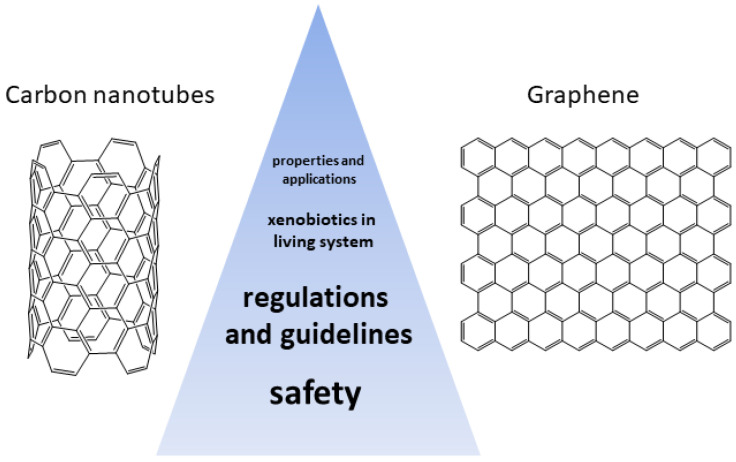
The importance of physicochemical characteristics in relation to safety considerations.

The safety of carbon nanotubes and graphene materials has been a topic of research and discussion in recent years. As these materials are increasingly used in various industries, it is essential to understand their potential health and safety risks (Figure 3). Graphene and its derivatives have a lower aspect ratio, larger surface area, and better dispersability in most solvents compared to carbon nanotubes [78]. These features theoretically offer significant advantages in terms of safety over the inhomogeneous dispersion of fiber-shaped carbon nanotubes. Graphene’s unique properties, such as its high surface area and high conductivity, make it a promising material for various applications, including electronics, energy storage, and biomedical devices. The direct comparison of graphene materials with carbon nanotubes regarding their safety and risk is challenging. However, a plethora of studies have reported the behavior of graphene [79,80,81,82,83,84,85,86,87,88,89,90,91] and carbon nanotubes [92,93,94,95,96,97,98] in in vitro and in vivo models, as well as their potential environmental hazards to humans.

More specifically, some studies have suggested that graphene may be less toxic than carbon nanotubes [99]. For example, one study found that graphene oxide was less toxic than multi-walled carbon nanotubes in human lung cells [100]. The safety risks associated with graphene materials entirely depend on the specific types of graphene materials and how they are investigated or applied. Therefore, generalizations about the toxicity of graphene should be made with caution. Carbon-based nanomaterials such as single-walled carbon nanotubes are susceptible to degradation via immune cells. This degradation can lead to the release of toxic substances, which may cause inflammation and damage to surrounding tissues. However, some studies have suggested that graphene may be less susceptible to immune cell degradation than carbon nanotubes. For example, graphene oxide was less susceptible to degradation by macrophages than single-walled carbon nanotubes. There are still challenges and limitations associated with the need for standardized methods to evaluate their toxicity. In vitro studies have shown that carbon nanotubes and graphene can cause oxidative stress, inflammation, and cell DNA damage [101]. However, these studies have limitations regarding their ability to predict the effects of these materials on living organisms [78]. In vivo studies revealed that exposure to carbon nanotubes can cause lung damage, inflammation, and fibrosis in animals (citations 2 to 7 in [102]). These research findings have not always been consistent, and there are still uncertainties about the long-term effects of exposure. We have only found a few ongoing and completed clinical trials regarding graphene- and carbon nanotube-based medical applications on clinicaltrials.gov. However, it is essential to note that human exposure to these materials is still relatively low, and the potential risks are mainly associated with occupational exposure [103], not environmental [104]. For instance, predicted environmental concentrations of carbon nanotubes in the air are between 0.001 and 0.008 ng/m${}^{3}$ [105], which is orders of magnitude lower than levels of benzo[*a*]pyrene (B*a*P) (a common and strong carcinogenic polycyclic aromatic hydrocarbon present worldwide, mainly in polluted cities) that can have concentrations up to 10–20 ng/m${}^{3}$ in outdoor air [106].

**Figure 4:**
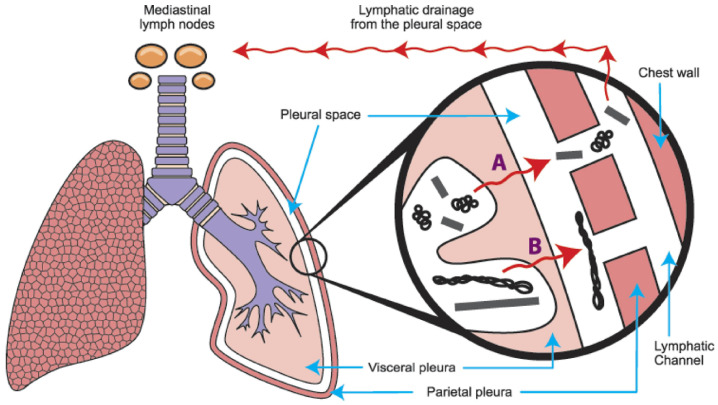
Length-dependent retention and possible clearance of CNTs from the lungs. Reprinted with permission from [107]. (Copyright 2011 published by Elsevier).

Furthermore, the properties of carbon nanotubes and graphene materials can vary depending on their size, shape, and surface chemistry, which makes it difficult to draw general conclusions about their safety. Nevertheless, there seems to be a consensus that long carbon nanotubes can act like asbestos and persist in the lungs causing inflammation and potentially long-term disease. In contrast, the fraction of shorter fibers can exit through the lymphatic drainage. This issue was studied, among others, by Murhpy et al. [107] (see for example Figure 4). The authors showed in a mouse model the length-dependent clearance of CNTs from the pleural space. Both short and long CNT fibers can deposit in the subpleural alveoli. However, short and small CNTs migrate to the pleural space, escaping in the flow of pleural fluid through the stomata, from where they follow the lymphatic drainage (marked by letter A in Figure 4). On the contrary, the longer fibers and CNTs are unable to pass through and are trapped, causing irritation and possibly long-term illness (depicted by B in Figure 4) [107].

Another study revealed that graphene oxide was more susceptible to neutrophil degradation than multi-walled carbon nanotubes. These findings suggest that graphene may have advantages over carbon nanotubes regarding immune cell degradation. As mentioned, carbon nanotubes and carbon nanofibers may pose a respiratory hazard. Inhalation of these materials can lead to lung damage, inflammation, and fibrosis. However, these materials’ toxicity depends on their size, shape, surface area and concentration.

**Figure 5:**
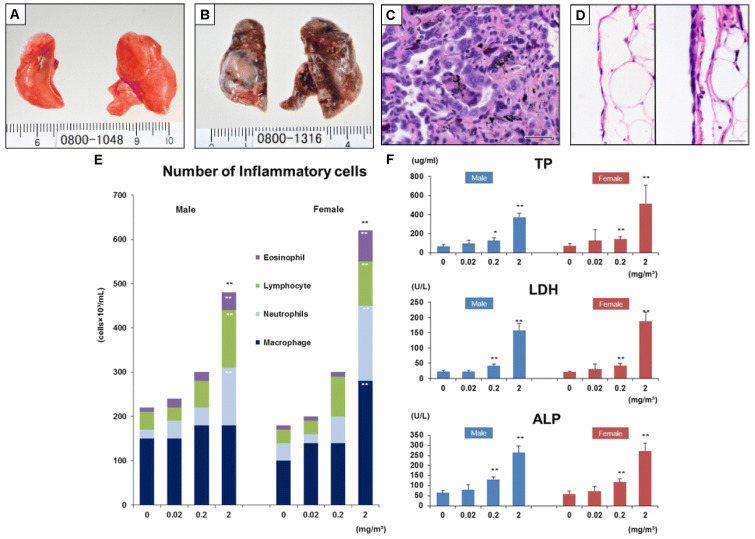
(**A**–**D**) Macroscopic and microscopic findings of the lungs and parietal pleura from a control male rat (**A** and left image of **D**) and a rat exposed to carbon nanotubes (the other images), respectively. (**E**,**F**) The results of cytological and biochemical analyses of the bronchoalveolar lavage fluid (BALF) of rats exposed to carbon nanotubes at different doses (X-axis) and clean air (control), respectively. (**E**) After being exposed to MWCNTs, both males and females experienced a concentration-dependent increase in the number of inflammatory cells, including eosinophils, lymphocytes, neutrophils, and macrophages. (**F**): TP—total protein, LDH—lactate dehydrogenase, ALP—alkaline phosphatase concentrations in the BALF; for male and female rats. Error bars corresponds to SD for 5 rats. All parameters are significantly increased in a concentration-dependent manner in males and females. For both (**E**,**F**) * represents *p* < 0.05 and ** signifies *p* < 0.01 by Dunnett’s multiple comparison test. Reprinted with permission from [108]. (Copyright Springer under Creative Commons Attribution 4.0 International License). scale bar = 25 μm.

**Figure 6:**
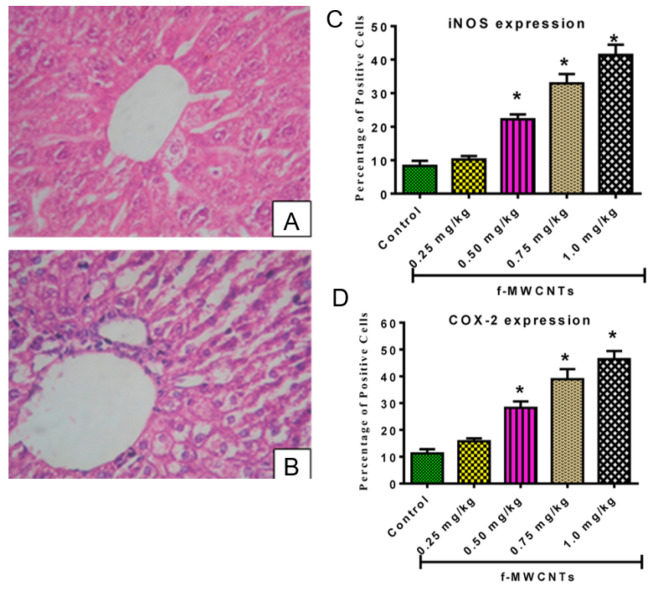
The hepatotoxicity of multi-walled carbon nanotubes. (**A**,**B**) Histopathological examination of a control (**A**) mouse and a mouse exposed to 100 mg/kg body weight MWCNTs at day 21 (**B**) [115]. Copyright 2013 published by Elsevier). (**C**,**D**) Analysis of pro-inflammatory enzyme expression show a dose-dependent increase in COX-2 and iNOS in the liver of carbon nanotube-treated rats compared to the control; *: values differ significantly from control (*p* < 0.05) as stated in [116]. (Adapted with permission from [115,116]. (Copyrights 2013 and 2018 respectively, published by Elsevier).

For instance, Kasai et al. performed an essential long carcinogenicity study using an animal model [108]. The rats were exposed to multi-walled carbon nanotubes for almost 2 years at different concentrations in the air (0.02, 0.2, and 2 mg/m${}^{3}$) for 6 h per day, five days a week. Interestingly, the study revealed a similar survival rate in both the control group (clean air) and rats exposed to MWCNTs, exceeding 72% in males and 68% in females at the end of the experimental period. There was no significant disagreement in the survival rates between the exposed and control groups. However, the rats exposed to the highest concentration of carbon nanotubes (2 mg/m${}^{3}$) developed bronchiolo-alveolar carcinoma (see Figure 5B), characterized by numerous visible white areas and nodules in the lungs. In contrast, the lungs of healthy control animals exposed to clean air are shown in Figure 5A. Histopathology (Figure 5C) confirmed these findings, revealing abnormal hyperplasia with proliferative fibrous connective tissue. Furthermore, the deposited multiwalled carbon nanotubes are visible as black spots/granules. Moreover, the polarity of the epithelial cells is poorly defined, and the nuclei of the cells differ in size. In Figure 5D, we can further compare normal parietal pleura (left image) to rats exposed to 2 mg/m${}^{3}$ (right side of Figure 5D), which indicates mesothelial hyperplasia of the parietal pleura. To complete the analysis, the authors carried out cytological and biochemical analyses of the bronchoalveolar lavage fluid (Figure 5E,F). There is a concentration-dependent relation between exposure to MWCNTs and the number of inflammatory cells. The higher the concentration of carbon nanotubes, the higher the number of eosinophils, lymphocytes, neutrophils, and macrophages, thus indicating an immune system response. In conjunction with the increase in inflammatory cells, the total protein (TP), lactate dehydrogenase (LDH), and alkaline phosphatase (ALP) levels, which are commonly used to asses liver function and toxicological effect, also increased in response to a greater dose of MWCNTs. In summary, the study by Kasai et al. showed that multi-walled carbon nanotubes were more toxic than single-walled carbon nanotubes in rat lungs. These findings highlight the importance of considering the specific properties of carbon nanotubes when assessing their toxicity. Another study found that graphene oxide was less toxic than single-walled carbon nanotubes in zebrafish embryos [109]. However, these findings are inconclusive, and more research is needed to fully understand the safety profiles of these materials. As mentioned above, specific studies report inhalation and lung homeostasis as the main predicted route of human exposure to carbon-based materials. Yet, since the majority of xenobiotic detoxification takes place in the liver (via CYP enzymes, including cytochrome P450, as well as glutathione S-transferases, and UDP-glucuronosyltransferases) [110,111,112,113], it is also essential to look at the hepatotoxicity of carbon nanotubes and graphene materials, especially in animal models. In a study involving Kunming mice, mice were intravenously injected with acid-oxidized and Tween-80-dispersed MWCNTs at 10 and 60 mg/kg body weigh for 15 and 60 days. The results showed that the mice experienced hepatotoxicity and altered gene expression of cytochrome P450 [114]. Another study explored the effects of MWCNTs on Swiss mice, where a single oral dose of MWCNTs at 60 and 100 mg/kg was administered, and the animals were investigated post exposure on days 7, 14, 21 and 28. In general, this led to decreased activities of superoxide dismutase (SOD) and catalase (CAT). MWCNTs caused hepatotoxicity, including inflammation, vacuolation of hepatocytes, hemorrhagic clots, and oxidative damage. However, this was only observed at the high dose level, and no significant changes in liver histology were observed at the lower dose level nor in the day 7 group (see Figure 6) [115]. Furthermore, a recent study by Adedara et al. showed a dose-dependent increase in hepatic oxidative stress, inflammation, and apoptosis in MWCNT-treated rat groups. The authors intraperitoneally administered the MWCNT suspension at the low doses of 0.25, 0.5, 0.75 and 1.0 mg/kg for five consecutive days and measured liver function and pro-inflammatory biomarkers [116]. Regarding graphene, a similar study to [115] was recently conducted on the hepatotoxicity of graphene oxide in Wistar rats [117]. Graphene was administered intraperitoneally at following doses: 0.4 mg/kg body weight (low dose), 2.0 mg/kg (mid dose), and 10.0 mg/kg (high dose). Of note, the applied low dose of graphene oxide did not result in liver inflammation. Significant hepatotoxicity, including elevated levels of alanine transaminase, alkaline phosphatase and catalase, as well as abnormal histological features were observed in the high dose group. These studies suggest that both carbon nanotubes and graphene oxide have substantial toxic potential on the mammalian liver, which increases with concentration and exposure time.

To give a simple overview of our current understanding of the safety of carbon nanotubes and graphene materials regarding their toxicity, biocompatibility, bio-persistence, and ways of modifying their effects as xenobiotics, we prepared the tables below. A summary of selected publications concerning graphene (Table 1) and carbon nanotubes (Table 2) is shown below. These tables aim to provide the reader with extra information to ponder, discuss, and consider while planning work or experiments with carbon-based nanomaterials.

**Figure 7:**
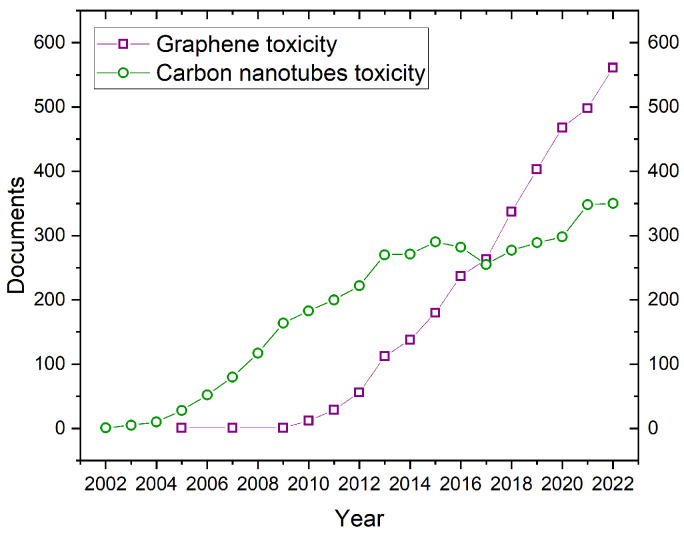
Results of the Scopus database search for “graphene toxicity” and “carbon nanotube toxicity”. A large increase in graphene toxicity studies after 2010—the Nobel Prize in Physics for graphene was awarded. For carbon nanotubes, from 2013 the number of papers on CNT toxicity remains consistent. For all the queries we searched within “Article title, Abstract, Keywords” and we used the logic operator “AND”. For instance, for graphene toxicity the query was as follows: “(TITLE-ABS-KEY (graphene) AND TITLE-ABS-KEY (toxicity))”. We also limited the search to all completed years. Thus, 2023 was not taken into consideration. The database was accessed on 12 October 2023.

The safety risk assessment of any new type of nanomaterial (such as graphene or carbon nanotubes) is challenging and economically expensive. The initial and most straightforward step scientists take is in vitro tests (using cell culture) in which certain cell lines are exposed to graphene or CNT materials. Standard assays, such as MTT, Live/Dead, or Alamar Blue, are used to assess cytotoxicity. This first screening provides potential answers regarding safety and toxicity, although animal models are necessary to establish real-life risks. Furthermore, the toxic response revealed in in vitro models may not be noticed in the animal model. In vitro models are generally less expensive and time-consuming than in vivo models. Researchers can use them to study a broader range of effects and have more control over the experimental conditions. On the other hand, in vitro studies are not always relevant to human health. It is challenging to extrapolate the results from in vitro studies to the human body. In vivo models are more often applicable to human health as they can provide more information about the effects of a substance on the whole body. However, in vivo models are more expensive and time-consuming than in vitro models. The best model type depends on the specific research question. Moreover, the likelihood of identifying immunotoxicity caused by nanomaterials increases from early in vitro models to preclinical in vivo and clinical phase studies. We recommend the comprehensive literature review on the correlation between in vitro and in vivo immunotoxicity tests by Marina A. Dobrovolskaia and Scott E. McNeil [127]. Lastly, it is important to point out that the use of animals models has raised several ethical questions such as the animal welfare, the possible alternatives, the moral considerations and regulations to name only a few [128,129,130]. However, efforts have been made to propose alternative solutions including computational and in silico methods [131,132]. Despite these differences and challenges, researchers are continuing to develop new and improved methods for determining nanomaterial toxicity, and we can expect to learn more about the safety of graphene in the upcoming years. As seen in Figure 7, the number of publications in the Scopus database tackling carbon nanotube toxicity has been established at a constant level from 2012. More precisely, the number of publications on CNT toxicity has been relatively constant over this time period (2012–2022), with a slight increase in recent years. A constant publication rate does not necessarily mean that a consensus has been reached on the toxicity of carbon nanotubes. It is possible that the number of publications has plateaued because more has been learned about their toxicity, and there is relatively limited new information to be discovered. In this regard, it is important to point out that in 2017, the IARC released a classification regarding the cancer risk of carbon nanotubes (MWCNTs and SWCNTs) [133]. In contrast, if we look at graphene toxicity studies, it distinctly grows from 2010 to 2017, the year the Nobel Prize was awarded for graphene discovery.

## 4. Regulation and Guidelines

**Figure 8:**
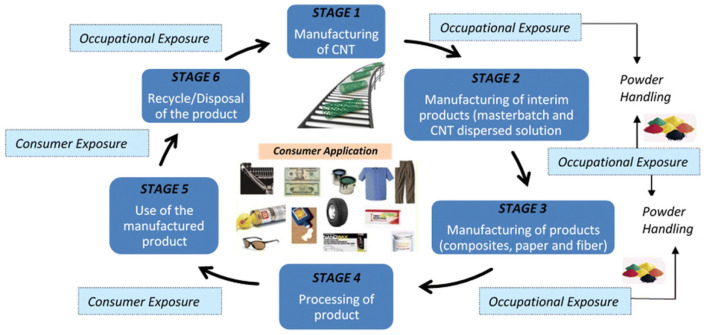
The importance of life cycle assessment for carbon nanotubes and graphene-based materials. (Reprinted with permission from [151]. Copyright Springer under Creative Commons Attribution 4.0 International License).

Several governmental entities have published guidelines concerning the occupational safety and handling of nanomaterials (Figure 8). For instance, the World Health Organization (WHO) has published Guidelines for the potential risks of manufactured nanomaterials [134]. Several recommendations are listed such as the assessment of the health hazards of nanomaterials, the assessment of the exposure to nanomaterials, the control of the exposure to nanomaterials, health surveillance, and the training and involvement of workers [135,136]. The Canadian government published in recent years a document concerning the health and safety considerations of engineered nanoparticles detailing the directions that need to be followed [137]. Similarly, the United Kingdom published guidelines on the use of carbon nanotubes and nanomaterials [138]. The guidelines list the legal duties (COSHH, DSEAR, REACH), the assessment and the control of risk. The United States, through the EPA (Environmental Protection Agency), also published a white paper on nanotechnology including a risk assessment of nanomaterials as well as recommendations for environmental applications, risk assessments, pollution prevention and environmental stewardship, collaborations, intra-agency workgroups and training [139]. At last, the government of Malaysia also published guideline documents concerning the handling of nanomaterials [140].

Likewise the occupational exposure of carbonaceous materials has also been examined and discussed by several studies in a manufacturing context [141,142,143,144,145]. In this regard, the National Institute for Occupation Safety and Health (NIOSH) published a bulletin reporting the occupational exposure to CNTs and nanofibers [146]. However, several studies carried out on animals showed cases of pulmonary fibrosis and inflammation. Likewise, recent studies have shown adverse health effects to humans for both CNTs and graphene materials [147,148]. Therefore, a series of recommendations have been proposed as follows: (1) recommendations for employers (medical screening and surveillance, worker education, periodic evaluation of data), and (2) recommendations for workers. In this vein, the Swedish government also published a report on the exposure toxicology and protective measures in the work environment [149]. Finally, the Australian government published guidelines on the safe handling and use of carbon nanotubes [146]. The risk management, including aspects such as detailed hazard analysis, exposure assessment, risk control and monitoring, are detailed in [77,150]. Specifically for graphene, the Swedish Chemical Agency together with SIO Grafen published a report describing an overview of the safety and regulation of graphene [146].

It is important to point out that carbon-based materials can be classified in terms of their carcinogenic risks to humans. The International Agency for Research on Cancer (IARC) analyzed carcinogenic hazards and published a series of monographs detailing the evaluation of carcinogenic risk of carbon materials [133].

Table 3 summarizes the IARC group of several carbon-based materials (agent). Carbon black and multi-walled carbon nanotubes (MWCNT-7) are both under the 2B group, referring to being possibly carcinogenic to humans. On the other hand, single-walled carbon nanotubes and multi-walled carbon nanotubes (other than MWCNT-7) are under group 3, i.e., not classifiable as carcinogenic to humans. We highlight that carbon materials were classified by the IARC recently in 2017, which shows that it can take about quarter of the century to obtain public recommendations (“consensus”) regarding the carcinogenic hazards of new nanomaterials—CNTs were discovered in 1991. Interestingly, the IARC classification was provided a few years after the number of articles in Scopus on CNT toxicity reached a plateau (see Figure 7). In addition, we recommend the works of the Morimoto, Fukushima and Barbarino groups, which have also delved into the carcinogenicity of CNTs [152,153,154]. At last, it is important to mention that, at the time of this review, there were no relevant studies concerning the carcinogenicity of graphene.

## 5. Conclusions

In summary, this review provides an overview of carbon nanotubes and graphene in relation to their impact on living systems. The initial section delved into the synthesis of carbon nanotubes and graphene, along with various techniques employed to analyze their physical and chemical attributes. Subsequently, the pathways of exposure to these materials, including inhalation, dermal contact, ingestion, and environmental exposure, were examined. The review then discussed the effects of carbon nanotubes and graphene on living systems, touching upon their current safety considerations. Finally, it highlighted the established guidelines and regulations governing their handling.

Now, to answer the initial question: carbon nanotubes and graphene materials as xenobiotics in living systems: Is there a consensus on their safety? Based on the information available, there is, for CNTs and derived materials, an agreement on their potential risk to human health and the environment. The NIOSH proposes a recommended exposure limit (REL) of 1 µg/m${}^{3}$ of elemental carbon as a respirable mass 8 h time-weighted average concentration [146]. Indeed, the latter agency reports that findings from recent animal experiments strongly suggest that carbon nanotubes (CNTs) and carbon nanofibers (CNFs) could potentially present respiratory risks. NIOSH still conducts ongoing studies to estimate this limit further. However, concerning graphene, intensive exposure assessment studies on its safety have yet to be completed by international or national agencies advising REL for graphene materials. Therefore, reaching a consensus about their safety in living systems is challenging. Regardless, it is essential to point out that the intricacies of comprehending their potential hazards have been compounded by several elements to account for, such as the variability in materials (CNTs or graphene, their sizes, their shapes, or their surface chemistry), the testing methods (in vivo or in vitro), and the exposure routes (inhalation, dermal contact, or digestion).

That said, to help the reader understand the issue, the following key elements must be considered when assessing the safety of CNTs and graphene in living systems: (1) The end application (CNTs and graphene can be used in a wide range of applications leading to various routes of exposure); and (2) the potential health risks (CNTs and graphene may have adverse effects of biological systems), and thus immune-suppressed models (in vivo and repeated-dose administration) should be considered; (3) the material variability (variations in size, shape and functionalization may affect its biocompatibility), and therefore the characterization techniques employed to identify and quantify its properties; (4) the lack of standardization (lack of standardized protocols and characterization methods); (5) the safety considerations (the published safety guidelines will likely evolve over time); and (6) the long-term studies (most published studies center around short-term effects; however, understanding the chronic exposure to CNTs and graphene accumulation over time is essential). In addition, taking the points listed above into consideration, a consecutive effort should be taken to establish standardized, interdisciplinary research approaches to categorize the safety of new materials when developing them.

Moreover, with the potential growing usage of graphene and carbon nanotubes in the future, several questions become pertinent: (a) How to safely store graphene used in electronics after its disposal? (b) What are the long-term effects of graphene and carbon nanotube wastes, for example, in the next 20–40 years? (c) What are the stability and toxic effects of graphene and carbon nanotubes in composites, especially polymeric one? (d) What would be the impact on living organisms beside humans (i.e., plants or bacteria)? (e) How should we encapsulate or coat carbon-based device components to reduce wear and enhance user safety [155,156,157,158,159,160]?

Finally, the implementation of long-lasting collaborations among scientists, regulatory bodies, and industry stakeholders is critical to address these challenges and provide clearer guidance on the safe use and potential mitigation strategies for any associated risks regarding CNTs and graphene. Similarly, because the field of nanotechnology is dynamic, with new discoveries and advancements occurring regularly, it is of the utmost importance that the latest research findings and developments are available for policymakers, researchers, and industry professionals to make informed decisions regarding the safe use of these materials, particularly in living systems.

## Figures and Tables

**Table 1:** Our current understanding of the safety of carbon nanotubes and graphene materials. A summary of selected publications regarding the toxicity, biodegradability and safety of graphene.

Reference, Year, Paper Type	Summary	What Was Tested and How?	Additional Comments
[118], 2014, review	Graphene, graphene oxide, and reduced graphene oxide elicit toxic effects both in vitro and in vivo. Surface modifications can significantly reduce their toxic interactions with living systems.	Discussed toxicological effects and potential toxicity mechanisms of graphene, graphene oxide, and reduced graphene oxide in bacteria and mammalian cells.	The generation of reactive oxygen species (ROS) is the most commonly acknowledged mechanism responsible for the toxicity induced by graphene nanomaterials in living systems. The toxicity of graphene nanomaterials in biological systems can be significantly influenced by their physicochemical properties (i.e., particle size, particulate form, surface functional groups, and oxygen content/surface charges).
[119], 2017, review	Graphene toxicity is a double-edged sword in terms of risks and opportunities. Authors confirm that the most recent research reports establish that graphene in any of its numerous forms and derivatives must be treated as a potentially hazardous material.	Analysis of the effects of graphene on microorganisms, protozoa, plants, invertebrates and lower vertebrates as well as in vitro and in vivo models.	The significant discrepancy and frequent controversy among experimental results, even within closely related models, emphasize the need for systematic, coordinated, multi-center research. This should include thorough physicochemical characterization of the graphene materials used in each study.
[120], 2018, original article	Graphene nanopores are likely to have a low bioavailability in lung cancer cells and rats. Graphene nanopores caused early apoptosis in both SKMES-1 and A549 lung cancer cells.	in vitro and in vivo studies, early and late apoptosis studies in SKMES-1 and A549 cells, sub chronic toxicity in rats intraperitoneally injected with graphene material, blood biochemistry, liver and kidney enzymes functions analysis, oxidative stress biomarkers, histological examinations.	We have described graphene nanopores as they are a relatively new derivative of graphene. The authors showed that cellular toxicity increases with the dose when studying single vs. multiple doses. However, an appropriate control is with reduced graphene oxide, which is the source material for nanopores.
[121], 2019, review	The biodegradability of flat nanomaterials is essential in living organisms. Oxidative enzymes (i.e., peroxidases) can catalyse the degradation of graphene oxide in test tubes, in vitro and in vivo. The biodegradation of both single- and few-layer graphene was proven.	Analysis of state-of-the-art publications concerning the biocompatibility and biodegradability of graphene-related materials.	Most of the research conducted so far has been in vitro studies, but it is crucial to expand critical validation tests to whole-model organisms/animal models.

**Table 2:** Our current understanding of the safety of carbon nanotubes and graphene materials. A nummary of selected publications regarding the toxicity, biodegradability and safety of carbon nanotubes (CNTs).

Reference, Year, Paper Type	Summary	What Was Tested and How?	Additional Comments
[122], 2006, original article	Multi-walled carbon nanotubes might be either toxic or non-toxic depending on the medium used to cultivate *Tetrahymena pyriformis*.	Experiments with various doses of multi-walled carbon nanotubes (MWNTs). Evaluated growth of *Tetrahymena pyriformis*, level of malondialdehyde, and superoxide dismutase activity.	Studies regarding the biological effects of the interaction of MWCNTs with certain ingredients of culture media would help us understand the mechanisms carbon nanotube toxicity to living systems.
[123], 2006, review	CNTs appear to possess a unique ability to stimulate mesenchymal cell growth and cause granuloma formation and fibrogenesis. In several studies, CNTs show more adverse effects than the same mass of NP carbon and quartz—used as a benchmark of particle toxicity.	Analysis of workplace safety based on the published toxicity of inhaled CNTs, mesenchymal cell growth, granuloma formation, fibrogenesis, oxidative stress, and inflammation.	CNTs should be considered in the same way as other bio-persistent fibers in workplace risk assessments. Therefore, similar similar control and assessment approaches should be taken.
[124], 2011, feature article	How to create low-toxic CNTs. The toxicity grade for a nanomaterial depends on more than ten factors (i.e., adsorbability, size, surface charge, or chemical modifications).	The analysis of biocompatibility, adme regulation, toxicity of carbon nanotubes, nanotoxicity mechanisms, and cellular and molecular interactions.	It is possible to alter the biological and toxicological properties of carbon nanomaterials by chemical modifications, therefore affecting the way in which these modified CNTs interact with biological systems.
[125], 2013, review	The underlying mechanisms of CNT toxicity include oxidative stress, inflammatory responses, malignant transformation, DNA damage and mutation, and the formation of granulomas and interstitial fibrosis.	Oxidative stress, inflammatory responses, malignant transformation, DNA damage and mutation (errors in chromosome number and disruption of mitotic spindles), formation of granulomas, and interstitial fibrosis. Reviewed data from several cell lines and animal models (rats, mice).	Researchers need to standardize their choices in terms of the cell line, animal species, and exposure conditions to ensure comparable results among different institutions and countries.
[126], 2022, review	CNTs located on a substrate had negligible impact, i.e., 90% of studies report good viability and cell behavior similar to the control; therefore, CNTs could be considered as a prospective conductive substrate for cell culture. CNTs are a promising platform for fundamental studies in targeted drug delivery, chemotherapy, tissue engineering, biosensing fields, etc.	Analysis of nearly 200 original publications regarding parameters such as toxicity doses, studied animal cell types, the impact of incubation time, applied toxicity tests, and viability.	Diameter, length, purification procedure, and synthesis may significantly affect CNT toxicity. The authors emphasize an urgent need for a straightforward, standardized, universal approach for the testing of the materials’ safety or toxic impact before conducting costly and time-consuming studies according to guidelines such as the OECD principles for proper laboratory practice.

**Table 3:** Classification of carbon nanotubes and graphene materials by The International Agency for Research on Cancer (IARC) [133].

Agent	IARC Group	Year	CAS
Carbon black	2B	2010	1333-86-4
CNTs, single-walled	3	2017	308068-56-6
CNTs, multi-walled, MWCNT-7	2B	2017	308068-56-6
CNsT, multi-walled, other than MWCNT-7	3	2017	308068-56-6
Graphene-based materials	—	—	7782-42-5

## Data Availability

Not applicable.

## Abbreviations

The following abbreviations are used in this manuscript:

CNTs	carbon nanotubes
CVD	chemical vapor deposition
SWCNTs	single-walled carbon nanotubes
MWCNTs	multi-walled carbon nanotubes
PAHs	polycyclic aromatic hydrocarbons
Ch	chiral vector
TEM	transmission electron microscopy
SEM	scanning electron microscopy
TGA	thermogravimetric analysis
GO	graphene oxide
AFM	atomic force microscopy
HR-TEM	high-resolution transmission electron microscopy
STEM	scanning transmission electron microscopy
DNA	deoxyribonucleic acid
COSHH	Control of Substances Hazardous to Health
DSEAR	Dangerous Substances and Explosive Atmospheres Regulations
REACH	Registration, Evaluation, Authorization and Restriction of Chemicals
EPA	Environmental Protection Agency
NIOSH	National Institute for Occupational Safety and Health
IARC	The International Agency for Research on Cancer
